# Comparison of manual chest compression versus mechanical chest compression for out-of-hospital cardiac arrest: A systematic review and meta-analysis

**DOI:** 10.1097/MD.0000000000037294

**Published:** 2024-02-23

**Authors:** Muhammad Omar Larik, Ayesha Ahmed, Moeez Ibrahim Shiraz, Seemin Afshan Shiraz, Muhammad Umair Anjum, Pratik Bhattarai

**Affiliations:** a Department of Medicine, Dow International Medical College, Karachi, Pakistan; b Department of Medicine, King Edward Medical University/Mayo Hospital, Lahore, Pakistan; c Department of Medicine, Mediclinic Parkview Hospital, Dubai, United Arab Emirates; d Department of Medicine, Dow Medical College, Karachi, Pakistan; e Department of Medicine, Manipal College of Medical Sciences, Pokhara, Nepal.

**Keywords:** cardiac arrest, manual chest compression, mechanical chest compression, OHCA, out of hospital cardiac arrest

## Abstract

**Background:**

Out-of-hospital cardiac arrest is a life-threatening condition that requires immediate intervention to increase the prospect of survival. There are various ways to achieve cardiopulmonary resuscitation in such patients, either through manual chest compression or mechanical chest compression. Thus, we performed a systematic review and meta-analysis to investigate the differences between these interventions.

**Methods:**

PubMed, Cochrane Library, and Scopus were explored from inception to May 2023. Additionally, the bibliographies of relevant studies were searched. The Cochrane Risk of Bias Tool for Randomized Controlled Trials, Newcastle-Ottawa Scale, and the Risk of Bias in Non-Randomized Studies-I tools were utilized to perform quality and risk of bias assessments.

**Results:**

There were 24 studies included within this quantitative synthesis, featuring a total of 111,681 cardiac arrest patients. Overall, no statistically significant differences were observed between the return of spontaneous circulation, survival to hospital discharge, short-term survival, and long-term survival. However, manual chest compression was associated with a significantly superior favorability of neurological outcomes (OR: 1.41; 95% CI: 1.07, 1.84; *P* = .01).

**Conclusion:**

Although there were no major differences between the strategies, the poorer post-resuscitation neurological outcomes observed in mechanical chest compression indicate the need for further innovation and advancements within the current array of mechanical devices. However, future high-quality studies are necessary in order to arrive at a valid conclusion.

## 1. Introduction

Out-of-hospital cardiac arrest (OHCA) remains a life-threatening condition that requires immediate intervention to increase the prospects of survival. It affects more than 356,000 people annually in the United States, with an undesirable survival rate of approximately 10%.^[1]^ Providing immediate and efficient cardiopulmonary resuscitation (CPR) can double or triple the likelihood of surviving an OHCA by providing temporary circulation to vital organs. Inefficient compressions and insufficient decompressions lead to decreased CPR efficiency. This directly affects the return of spontaneous circulation (ROSC) and long-term outcomes such as survival.^[2,3]^ Studies have debated whether the utilization of manual CPR increases the likelihood of survival as opposed to mechanical CPR.^[4,5]^ There are various approved devices on the market, namely “AutoPulse” and “Lund University Cardiopulmonary Assist System” (LUCAS), with a large number of studies performed comparing the efficacy of such devices. On account of the number of studies available, the conflicting results of such studies raise hesitancy and reluctance with respect to the superiority of either manual or mechanical chest compression.

LUCAS, introduced in 2002, remains a prominent design among the series of mechanical compressive devices available on the market. It provides resuscitation via piston-like motions on the chest of a patient, yielding a similar effect to that of manual chest compression. Load-distributing band cardiopulmonary resuscitation devices are another type of mechanical chest compression devices that utilize an electric motor to provide rhythmic motions on the chest of a patient, as opposed to the traditional piston-based mechanism. The use of mechanical chest compression devices has persistently remained controversial with respect to efficacy outcomes; however, the consistency and convenience of such devices are undeniable.

In light of these controversies, we conducted a comprehensive systematic review and meta-analysis to compare the usage of both manual chest compression and mechanical chest compression for various clinical cardiovascular outcomes, along with prespecified subgroup analyses performed based on the type of device (LUCAS, AutoPulse, and others) and the study design (randomized, prospective observational, and retrospective observational).

## 2. Methodology

### 2.1. Data sources and search strategy

This systematic review and meta-analysis was performed in consistency with the “Preferred Reporting Items for Systematic Review and Meta-Analyses” guidelines.^[6]^ Databases were comprehensively searched from inception to May 2023 for potentially relevant studies, including PubMed/MEDLINE, Cochrane Library, and Scopus. Additionally, the bibliographies of all potentially relevant studies were explored for further data. The inclusive search strategy employed for each database is available in Table S1, Supplemental Digital Content, http://links.lww.com/MD/L724. In order to utilize a rigorous methodology within this meta-analysis, an evaluation using the “Assessing the Methodological Quality of Systematic Reviews” (AMSTAR-2) guidelines was performed.^[7]^

### 2.2. Study selection and eligibility criteria

The relevant articles extracted via the comprehensive systematic search were exported to EndNote Reference Library, version X8.1 (Clarivate Analytics), wherein the duplicate articles were removed from study inclusion. A title-and-abstract-level search was performed by 2 independent investigators (M. O. L. and M. I. S.), followed by a full-text review of all shortlisted articles. In case of any discrepancies, a third investigator (A. A.) was involved in order to resolve any disputes between the independent investigators. Prespecified eligibility criteria were established in order to facilitate the inclusion of relevant studies for the systematic review, with the conditions of (i) studies reporting patients of out-of-hospital cardiac arrest, (ii) studies reporting comparisons of manual versus mechanical chest compressions, (iii) studies reporting at least one of the outcomes of interest, and (iv) published studies that were either randomized, non-randomized, prospective, or retrospective in nature. All other article types (e.g., letter to the editors reporting unoriginal data, case reports, systematic reviews, and narrative reviews) were excluded. For meta-analytical purposes, both randomized and non-randomized studies were aggregated, with no discrimination, in order to employ a greater patient sample size, as this approach yielded a significantly larger sample size in comparison to a randomized-only approach. No distinction was enforced on the type of mechanical device being used for mechanical compression throughout the study selection process.

### 2.3. Data extraction and quality of assessment

The following data were extracted: (i) baseline characteristics of included study population, (ii) return of spontaneous circulation, (iii) survival to hospital discharge, (iv) survival to hospital discharge with favorable neurological outcomes, or Cerebral Performance Category of ≤2, (v) survival for up to 24 hours, and (vi) survival for up to 30 days. Risk of bias and quality assessment were performed by 2 independent reviewers (A. A. and M. O. L.) via the Newcastle-Ottawa Scale (NOS) for Quality Assessment of Cohort Studies^[8]^ and the Cochrane Risk of Bias Tool for Randomized Controlled Trials.^[9]^ All studies were scored out of 9, with scores beyond 7 considered to have high quality and a low risk of bias. In case of any discrepancies, a third reviewer (M. I. S.) was involved in order to resolve any disputes between the independent reviewers.

### 2.4. Statistical analysis

All statistical analyses were performed using Review Manager software (RevMan version 5.3; Copenhagen: The Nordic Cochrane Center, The Cochrane Collaboration, 2014). The Mantel-Haenszel and random-effects models were used, with all dichotomous outcomes compared using odds ratios. Any *P*-values of ≤.05 were considered to be significant in all aspects of the analysis. Heterogeneity was measured and assessed via the Higgins I^2^ value, in which values <25% are not considered heterogeneous, values ranging from 25% to 75% are considered moderately heterogeneous, and any values >75% are considered to be significantly heterogeneous.^[10]^ A pre-specified subgroup analysis was conducted for various mechanical chest compression devices, including LUCAS, AutoPulse, and others. Additionally, in cases of significant heterogeneity, an additional subgroup analysis of the study design (i.e., randomized, non-randomized, prospective observational, or retrospective observational) was performed in order to explore the sources of bias within our analysis.

## 3. Results

### 3.1. Literature search, characteristics of studies, and quality of assessment

**Figure 1.**
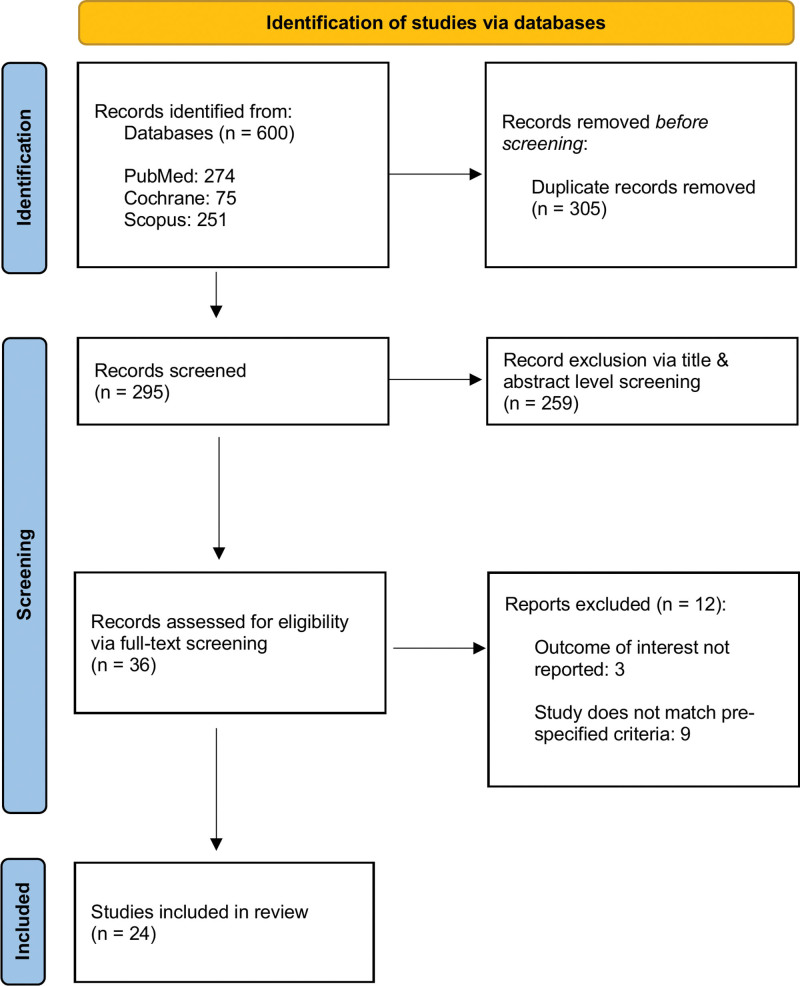
PRISMA flowchart. PRISMA = Preferred Reporting Items for Systematic Review and Meta-Analyses.

The initial search yielded a total of 600 studies up to May 2023. After the removal of duplicate studies and a comprehensive screening process, there were 36 studies shortlisted for full-text screening. Conclusively, there were 24 studies shortlisted for inclusion within this systematic review and meta-analysis.^[4,5,11–32]^ The results of the detailed screening process can be observed in the Preferred Reporting Items for Systematic Review and Meta-Analyses flowchart, illustrated in Figure 1. There were a total of 111,681 patients included in this extensive systematic review and meta-analysis, with 83,599 patients receiving manual chest compression and 28,082 patients receiving mechanical chest compression.

The risk of bias and quality assessment of included studies was performed using the Cochrane Risk of Bias Tool for Randomized Controlled Trials, version 2.0, the NOS for Cohort and Case-Control Studies (NOS), and the Risk of Bias in Non-Randomized Studies-I tool.^[8,9]^ In total, there were 8 randomized controlled trials (RCTs) (including 4 cluster RCTs and 4 individual RCTs), 15 observational studies (including 13 cohort studies and 2 case-control studies), and a single non-randomized clinical trial. Overall, the RCTs were generally of moderate quality, the cohort/case-control studies were generally of high quality, and the non-RCT was of moderate quality. Detailed judgements and results of Randomized Controlled Trials, version 2.0, NOS, and Risk of Bias in Non-Randomized Studies-I have been highlighted in Table S2, Supplemental Digital Content, http://links.lww.com/MD/L725, Table S3, Supplemental Digital Content, http://links.lww.com/MD/L726, and Table S4, Supplemental Digital Content, http://links.lww.com/MD/L727, respectively.

The relevant patient baseline characteristics of the included study population are outlined in Table 1.

**Table 1:** Baseline characteristics of included study population.

Study	Study design	Duration of study	Device used	Participants, n		Mean age, y (SD)		Male, n		Shockable rhythm, n (%)		Witnessed, n (%)		Bystander CPR, n (%)		Epinephrine, n (%)	
				Mechanical	Manual	Mechanical	Manual	Mechanical	Manual	Mechanical	Manual	Mechanical	Manual	Mechanical	Manual	Mechanical	Manual
Ong (2006)^[4]^	Prospective	2001–2005	AutoPulse	284	499	67 (51–83)	68 (52–80)	161 (57)	269 (54)	65 (23)	102 (20)	94 (34)	172 (35)	85 (31)	158 (32)	–	–
Rubertsson (2014)^[5]^	RCT	2008–2013	LUCAS	1300	1289	69 (16–100)	69 (15–99)	869 (67)	857 (66)	374 (29)	383 (30)	861 (66)	840 (65)	745 (57)	709 (55)	–	–
Newberry (2018)^[11]^	Retrospective	2013–2015	LUCAS	763	2236	64 (47–81)	64 (47–81)	436 (57)	1347 (60)	94 (12)	300 (14)	279 (37)	959 (43)	337 (44)	828 (37)	754 (99)	2155 (96)
Zeiner (2015)^[12]^	Prospective	2013–2014	LUCAS, AutoPulse	283	655	63 (54–73)	70 (60–81)	205 (72)	390 (60)	93 (34)	133 (22)	158 (56)	354 (54)	153 (54)	293 (45)	–	–
Savastano (2019)^[13]^	Retrospective	2015–2017	AutoPulse	235	1166	63 (52–71)	80 (68–86)	197 (83)	647 (55)	100 (43)	160 (14)	203 (86)	819 (70)	121 (51)	351 (30)	–	–
Gonzales (2018)^[14]^	Retrospective	2016–2016	LUCAS	227	217	65 (55–77)	64 (52–76)	142 (63)	130 (60)	37 (16)	41 (19)	82 (36)	99 (46)	88 (39)	84 (39)	223 (98)	207 (95)
Chen (2021)^[15]^	Retrospective	2018–2020	LUCAS	279	273	77 (65–86)	78 (63–85)	154 (55)	152 (56)	73 (26)	58 (21)	135 (48)	146 (53)	197 (71)	131 (48)	15 (5)	10 (4)
Mistraletti (2022)^[16]^	Retrospective	2013–2016	LUCAS	305	1061	60 (50–68)	63 (52–70)	248 (81)	768 (73)	146 (48)	296 (28)	289 (95)	1015 (96)	153 (51)	348 (33)	–	–
Tantarattanapong (2022)^[17]^	Retrospective	2017–2019	LUCAS	34	193	59 (51–67)	68 (54–79)	25 (74)	119 (62)	9 (27)	41 (21)	25 (74)	168 (87)	8 (24)	97 (50)	–	–
Lin (2014)^[18]^	Retrospective	2010–2013	-	216	188	66 (47–85)	65 (48–82)	133 (62)	120 (64)	18 (8)	13 (7)	–	–	–	–	–	–
Mastenbrook (2020)^[19]^	Retrospective	2011–2017	LUCAS	80	110	66 (49–83)	66 (51–81)	46 (58)	69 (63)	13 (16)	10 (10)	35 (44)	57 (55)	0 (0)	1 (1)	–	–
Hayashida (2017)^[20]^	Prospective	2012–2013	-	918	5619	75 (63 − 83)	75 (63 − 83)	583 (64)	3375 (60)	77 (8)	397 (7)	498 (54)	2653 (47)	389 (42)	1870 (33)	175 (19)	1135 (20)
Jennings (2012)^[21]^	Case-control	2006–2010	AutoPulse	66	220	69 (53–78)	71 (55–78)	36 (55)	135 (61)	20 (30)	78 (36)	39 (59)	133 (60)	33 (54)	127 (62)	–	–
Buckler (2016)^[22]^	Retrospective	2013–2015	–	17,625	63,056	62 (52–75)	62 (52–75)	–	–	–	–	7526 (43)	27,934 (44)	–	–	–	–
Casner (2005)^[23]^	Case-control	2003–2003	AutoPulse	124	138	68 (52–84)	68 (53–85)	78 (63)	75 (54)	41 (33)	39 (28)	–	–	–	–	–	–
Anantharaman (2017)^[24]^	Cluster RCT	2011–2012	LUCAS	302	889	66 (51–81)	67 (51–83)	221 (73)	571 (64)	571 (64)	164 (18)	185 (61)	470 (53)	96 (32)	284 (32)	–	–
Wik (2014)^[25]^	RCT	2009–2011	AutoPulse	2099	2132	66 (50–82)	66 (50–82)	1295 (61)	1315 (61)	451 (21)	519 (24)	785 (37)	785 (37)	1024 (47)	1035 (49)	1958 (93)	1946 (91)
Hallstrom (2006)^[26]^	Cluster RCT	2004–2005	AutoPulse	394	373	67 (51–83)	66 (51–81)	252 (64)	245 (66)	119 (32)	122 (31)	175 (44)	181 (49)	127 (32)	132 (35)	283 (89)	265 (83)
Goa (2016)^[27]^	Cluster RCT	2011–2012	AutoPulse	69	64	63 (48–78)	64 (51–77)	50 (73)	44 (69)	9 (13)	8 (12.5)	46 (67)	38 (59)	–	–	53 (77)	56 (88)
Günaydin (2015)^[28]^	RCT	2005–2005	CardioPump	95	86	67 (53–81)	70 (56–84)	51 (54)	48 (56)	40 (42)	40 (47)	–	–	–	–	–	–
Perkins (2015)^[29]^	Cluster RCT	2010–2013	LUCAS	1652	2819	71 (55–87)	72 (56–88)	1039 (63)	1774 (63)	376 (23)	615 (22)	1001 (61)	1749 (62)	716 (43)	1238 (44)	–	–
Smekal (2011)^[30]^	RCT	2005–2007	LUCAS	75	73	69 (53–85)	71 (55–87)	50 (68)	50 (68)	20 (27)	20 (27)	50 (68)	53 (74)	25 (34)	22 (31)	–	–
Satterlee (2013)^[31]^	Retrospective	2008–2010	LUCAS	498	74	64 (51–76)	66 (53–80)	346 (70)	53 (72)	102 (21)	13 (18)	213 (43)	31 (42)	–	–	475 (96)	31 (43)
Axelsson (2006)^[32]^	Non-RCT	2003–2005	LUCAS	159	169	71 (58–84)	71 (58–84)	100 (63)	108 (64)	48 (30)	54 (32)	–	–	72 (45)	71 (42)	–	–

CPR = cardiopulmonary resuscitation, n = number of patients, N-RCT = non-randomized clinical trial, RCT = randomized controlled trial, SD = standard deviation, y = years.

### 3.2. Return of spontaneous circulation

**Figure 2.**
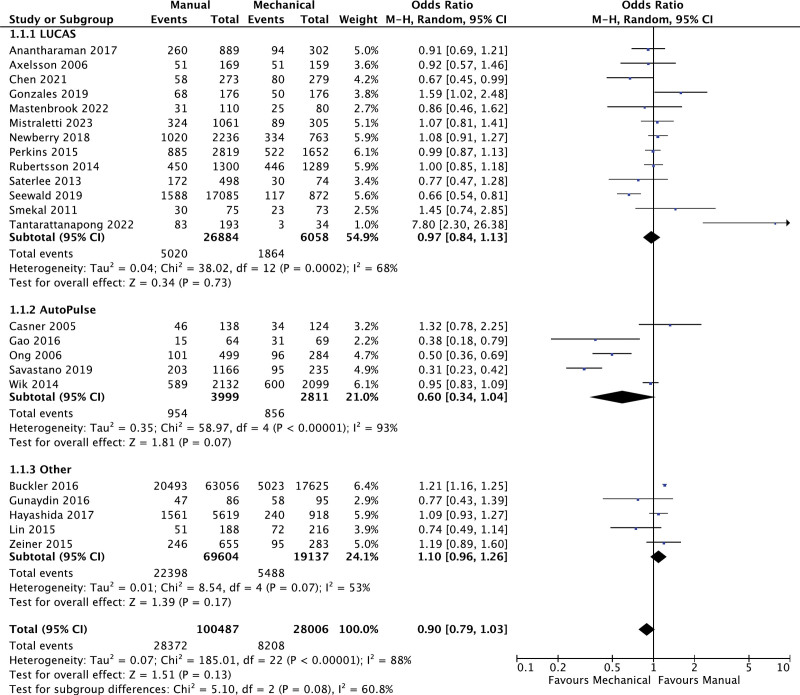
Forest plot for comparison of return of spontaneous circulation.

There were 23 studies comparing the effects of manual chest compression and mechanical chest compression on the return of ROSC. As per the analysis, there were no significant differences observed in ROSC (OR: 0.90; 95% CI: 0.79, 1.03; *P* = .13; I^2^ = 88%; Fig. 2). Moreover, there were no significant subgroup differences observed within this analysis (*P* = .08). AutoPulse performed marginally superior to LUCAS; however, this failed to reach any significant differences. In order to address the significant heterogeneity within this outcome, the subgroup analyses revealed that AutoPulse (I^2^ = 93%), prospective observational (I^2^ = 90%), and retrospective observational (I^2^ = 91%) were denoted as significant sources of heterogeneity. Although exclusion does not result in the eradication of heterogeneity, it was decreased to a moderate level (Fig. S1, Supplemental Digital Content, http://links.lww.com/MD/L728).

### 3.3. Survival to hospital discharge

**Figure 3.**
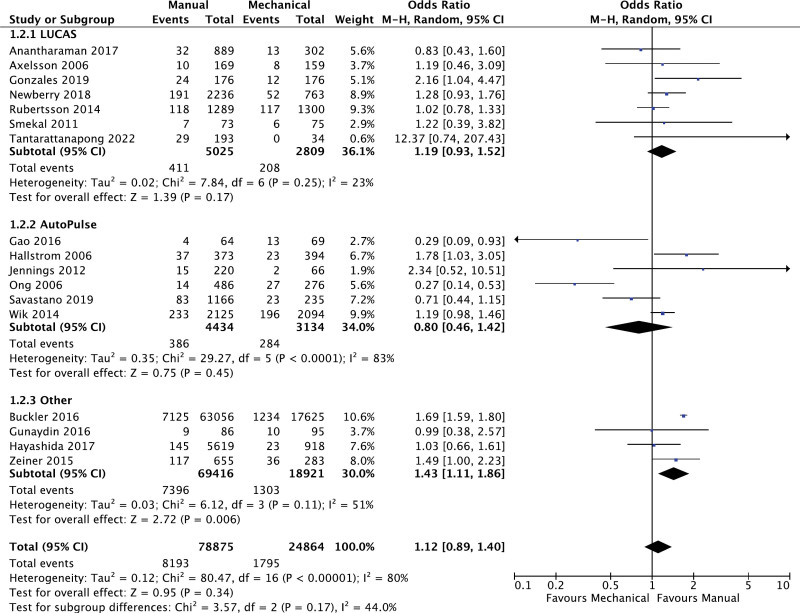
Forest plot for comparison of survival to hospital discharge.

There were 17 studies comparing the effects of manual versus mechanical chest compression on the rate of survival to discharge. As per the analysis, there were no significant differences observed in the rate of survival to discharge (OR: 1.12; 95% CI: 0.89, 1.40; *P* = .34; I^2^ = 80%; Fig. 3). Moreover, there were no significant subgroup differences observed within this analysis (*P* = .17). AutoPulse performed marginally superior to LUCAS; however, this failed to reach any significant differences. In order to address the significant heterogeneity within this outcome, the subgroup analyses revealed that AutoPulse (I^2^ = 83%) and prospective observational (I^2^ = 85%) were denoted as significant sources of heterogeneity. Although exclusion does not result in the eradication of heterogeneity, it was decreased to a moderate level (Fig. S2, Supplemental Digital Content, http://links.lww.com/MD/L729).

### 3.4. Favorable neurological outcomes

**Figure 4.**
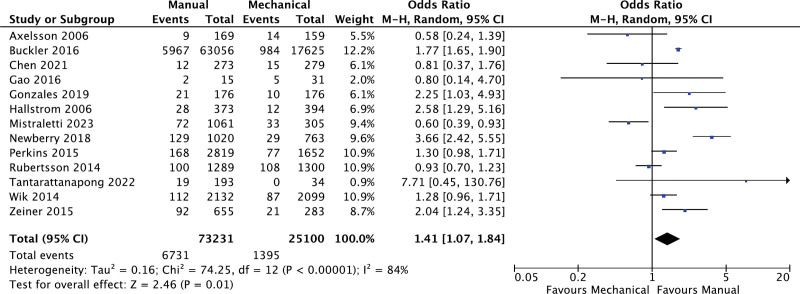
Forest plot for comparison of favorable neurological outcomes.

There were 13 studies comparing the effects of manual versus mechanical chest compression on the probability of favorable neurological outcomes after hospital discharge. As per the analysis, the cohort undergoing manual compression demonstrated significantly favorable neurological outcomes when compared to mechanical compression (OR: 1.41; 95% CI: 1.07, 1.84; *P* = .01; I^2^ = 84%; Fig. 4). Subgroup analyses were not considered, due to the limited number of studies reporting this outcome.

### 3.5. Short-term and long-term survival

**Figure 5.**
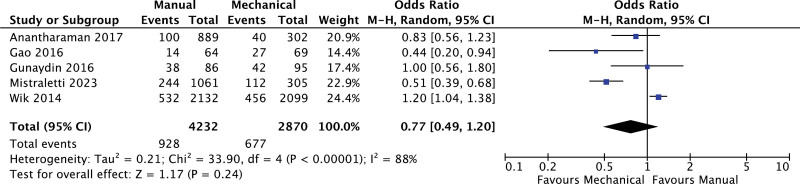
Forest plot for short-term survival (24 hours).

**Figure 6.**
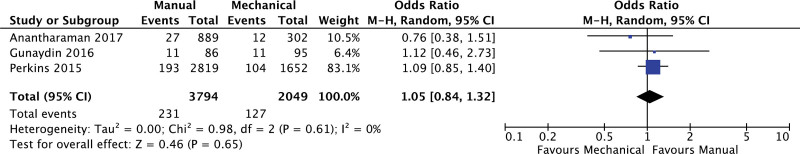
Forest plot for long-term survival (30 days).

There were 5 studies comparing the effects of manual versus mechanical chest compression on survival after 24 hours. As per the analysis, there were no significant differences observed in short-term survival (OR: 0.77; 95% CI: 0.49, 1.20; *P* = .24; I^2^ = 88%; Fig. 5). Moreover, there were 3 studies comparing the effects of manual versus mechanical chest compression on survival after 30 days. As per the analysis, there were no significant differences observed in long-term survival (OR: 1.05; 95% CI: 0.84, 1.32; *P* = .65; Fig. 6). Subgroup analyses could not be performed due to the limited number of studies reporting these outcomes. In conclusion, this meta-analysis highlighted several important findings. It was observed that the return of spontaneous circulation, survival to hospital discharge, short-term survival, and long-term survival did not significantly differ when manual chest compression was compared to mechanical chest compression. Greater innovation and technological advancements are encouraged with respect to mechanical chest compression devices. Further comprehensive studies, with greater sample sizes and more robust methodologies, are imperative in order to arrive at a robust conclusion.

## 4. Discussion

To the best of our knowledge, this systematic review and meta-analysis features the greatest number of studies and the largest patient population of any existing research comparing manual versus mechanical chest compressions in OHCA patients. This analysis featured a total of 24 included studies, with a total of 83,599 patients undergoing manual compression and 28,082 patients undergoing mechanical compression, resulting in the pooling of a total of 111,681 patients in this meta-analysis. Overall, there were no significant differences observed between manual versus mechanical chest compressions with respect to ROSC, survival to discharge, short-term survival (24 hours), and long-term survival (30 days). However, manual chest compression was significantly associated with better neurological outcomes when compared to mechanical chest compression.

Despite monumental advances in the field of cardiovascular medicine, the success rate in the management of OHCA remains undesirably low. One of these advances includes the development of mechanical chest compression and decompression devices that perform CPR on the cardiac arrest patient, such as LUCAS or AutoPulse. Mechanical devices provide consistent, high-quality chest compressions to OHCA patients in a manner that may never be achieved by standard manual compressions that are routinely administered in clinical practice.^[33]^ This led to a significant clinical and research attraction for the potential use of mechanical devices in OHCA patients. Additionally, the use of mechanical chest compression is often seen as superior in certain circumstances in which manual CPR cannot be safely delivered, such as transport, certain cardiovascular procedures, including percutaneous coronary intervention and bridge-to-invasive procedures such as extracorporeal circulation.^[34]^ However, mechanical devices have major drawbacks that limit their widespread use in emergency settings across the world. Firstly, the LUCAS-2 device is designed to perform in accordance with the American Heart Association 2005 guidelines, maintaining a chest compression depth of 5 cm.^[35]^ In the newer American Heart Association guidelines, it is recommended to have a minimum of 5 cm, with no maximal chest compression depth assigned.^[36]^ The rationale behind the increase in compression depth is attributed to an increase in systemic blood flow, leading to a proportionally increased blood flow to vital organs, including the heart and brain, which are necessary for a successful resuscitation.^[37]^ Thus, an updated mechanical device, with the ability to increase compression depth is necessary in order to ensure compliance with such guidelines. Moreover, mechanical chest compression has also been linked to poorer hemodynamic efficacy, as denoted by suboptimal values of _pet_CO_2_ and aortic pressures, which improved after switching to manual compression.^[37]^

There have been various studies conducted in the past that align with the results of this systematic review and meta-analysis. In a meta-analysis conducted by Zhu et al, both observational and RCTs did not demonstrate any significant differences between mechanical and manual compression for ROSC, survival to discharge, and favorable neurological outcomes.^[38]^ Although the results of this analysis were similar to the findings within our analysis, the authors also revealed the lack of significance of study type, as both observational studies and RCTs demonstrated similar metrics in all aspects. Additionally, in a meta-analysis conducted by Liu et al, which exclusively compared LUCAS devices with manual devices, a similar trend was observed with respect to ROSC, survival to discharge, and 30-day survival.^[39]^ Interestingly, in a meta-analysis conducted by Gao et al, the safety of manual versus mechanical chest compressions was compared.^[40]^ Although both strategies failed to reach significant differences with respect to the incidence of life-threatening injuries, the patients receiving manual chest compression were subject to a lower likelihood of posterior rib fractures and visceral (heart and lung) lesions. This finding further adds to the list of major drawbacks with respect to the use of mechanical devices in contrast with standard manual compression.

### 4.1. Clinical implications of the results

There are several implications arising from the results of our meta-analysis. Firstly, this meta-analysis provides contradictory results for the favorability of neurological outcomes in comparison with existing meta-analyses.^[38]^ As per existing analyses, there were no differences observed in neurological outcomes when manual chest compression was compared to mechanical chest compression. However, this analysis revealed superior neurological outcomes when OHCA patients were subjected to manual chest compression. This contradiction could be attributed to several factors, including the marginally larger sample size included in this analysis (111,681 patients versus 91,335 patients in the existing meta-analysis) or the separate pooling of RCTs and observational studies in the existing meta-analysis.^[38]^ Nonetheless, the suspected lack of effectiveness of mechanical devices in providing favorable neurological outcomes must be addressed. Firstly, this may be attributed to the outdated configuration being used within LUCAS and AutoPulse devices, which highlights the need for updated devices with the opportunity to provide deeper compression depths when needed, as opposed to the borderline 5-cm compressions being provided by current in-use devices. This may enhance systemic circulation and hemodynamics, leading to improved cerebral circulation and a subsequent improvement in neurological outcomes. Although mechanical devices are associated with superior hemodynamics, the failure to observe superior clinical outcomes may be attributed to the poorer safety profile associated with the use of such mechanical devices, which may potentially increase the risk of abdominal and thoracic injuries.^[40]^ Moreover, the possible delay within implementation of the mechanical device (i.e., pauses within chest compressions whilst deploying the respective device), in an emergency setting, may once again contribute to the failure of superior clinical outcomes observed. This can be exemplified by the increased time-to-defibrillation observed in certain included studies.^[5,25]^ Ultimately, there is an urgent indication for further comprehensive research within the exact science of mechanical devices in order to address these potential failures in an effective manner.

### 4.2. Strengths and limitations of this study

In comparison with previous meta-analyses conducted on this topic, the power of this study is undeniably enhanced due to the inclusion of the most recent studies, paired with an immensely enhanced patient data pool. Moreover, this study adds significant value to the existing literature by performing a pre-specified subgroup analysis of both LUCAS and AutoPulse devices, which has not been explored previously, to the best of our knowledge. This enables a direct comparison of efficacy between both of these popular and on-the-market devices. However, while the results of our analysis have been greatly supported by previous studies, it is imperative to highlight the limitations that occurred within the conduct of this study, which may contribute to significant bias. Firstly, although both LUCAS and AutoPulse were featured, there were no further subgroup analyses performed assessing the differences in efficacy between LUCAS-1, LUCAS-2, and LUCAS-3 devices. Secondly, this analysis featured ROSC in multiple forms, including prehospital, sustained, and undefined. This may possibly introduce bias within the results of this analysis, as sustained ROSC is far more difficult and unlikely to achieve in comparison with prehospital ROSC. Furthermore, certain included studies reported uncertainty within their retrospective data with respect to the potential inclusion of undocumented use of mechanical chest compression devices in the manual cohort, leading to incalculable inaccuracies within the results of this analysis as a result of information bias. Finally, the literature search spanning 3 databases and the bibliographies of all potentially relevant studies was not exhaustive, leading to the possibility of missed publications within the systematic search.

## 5. Conclusion

In conclusion, this meta-analysis highlights several important findings. It was observed that the return of spontaneous circulation, survival to hospital discharge, short-term survival, and long-term survival did not significantly differ when manual chest compression was compared to mechanical chest compression. Greater innovation and technological advancements are encouraged with respect to mechanical chest compression devices. Further comprehensive studies, with larger sample sizes and more robust methodologies, are imperative in order to arrive at a robust conclusion.

## Author contributions

**Conceptualization:** Muhammad Omar Larik.

**Formal analysis:** Muhammad Omar Larik, Ayesha Ahmed.

**Methodology:** Muhammad Omar Larik, Moeez Ibrahim Shiraz.

**Supervision:** Seemin Afshan Shiraz, Muhammad Umair Anjum, Pratik Bhattarai.

**Writing – original draft:** Muhammad Omar Larik, Moeez Ibrahim Shiraz.

**Writing – review & editing:** Muhammad Omar Larik, Seemin Afshan Shiraz, Muhammad Umair Anjum, Pratik Bhattarai.

## Supplementary Material












