# Supplementary material for: Comparison of manual chest compression versus mechanical chest compression for out-of-hospital cardiac arrest: A systematic review and meta-analysis
Source: Medicine (Baltimore). 2024 Feb 23;103(8):e37294. doi: 10.1097/MD.0000000000037294 (PMC10883626; doi:10.1097/MD.0000000000037294)
Supplement: Supplementary file 1 [file medi-103-e37294-s001.docx]

| **Search Strategy** | **Database** | **No. of Citations** |
| --- | --- | --- |
| ((("manual s"[All Fields] OR "manualization"[All Fields] OR "manualized"[All Fields] OR "manually"[All Fields] OR "manuals as topic"[MeSH Terms] OR ("manuals"[All Fields] AND "topic"[All Fields]) OR "manuals as topic"[All Fields] OR "manual"[All Fields] OR "manuals"[All Fields]) AND ("chested"[All Fields] OR "thorax"[MeSH Terms] OR "thorax"[All Fields] OR "chest"[All Fields] OR "chests"[All Fields]) AND ("compress"[All Fields] OR "compressed"[All Fields] OR "compresses"[All Fields] OR "compressibilities"[All Fields] OR "compressibility"[All Fields] OR "compressible"[All Fields] OR "compressing"[All Fields] OR "compression"[All Fields] OR "compression s"[All Fields] OR "compressions"[All Fields] OR "compressive"[All Fields] OR "compressively"[All Fields])) OR (("manual s"[All Fields] OR "manualization"[All Fields] OR "manualized"[All Fields] OR "manually"[All Fields] OR "manuals as topic"[MeSH Terms] OR ("manuals"[All Fields] AND "topic"[All Fields]) OR "manuals as topic"[All Fields] OR "manual"[All Fields] OR "manuals"[All Fields]) AND ("cardiopulmonary resuscitation"[MeSH Terms] OR ("cardiopulmonary"[All Fields] AND "resuscitation"[All Fields]) OR "cardiopulmonary resuscitation"[All Fields] OR "cpr"[All Fields])) OR (("manual s"[All Fields] OR "manualization"[All Fields] OR "manualized"[All Fields] OR "manually"[All Fields] OR "manuals as topic"[MeSH Terms] OR ("manuals"[All Fields] AND "topic"[All Fields]) OR "manuals as topic"[All Fields] OR "manual"[All Fields] OR "manuals"[All Fields]) AND ("cardiopulmonary resuscitation"[MeSH Terms] OR ("cardiopulmonary"[All Fields] AND "resuscitation"[All Fields]) OR "cardiopulmonary resuscitation"[All Fields])) OR (("conventional"[All Fields] OR "conventionals"[All Fields]) AND ("cardiopulmonary resuscitation"[MeSH Terms] OR ("cardiopulmonary"[All Fields] AND "resuscitation"[All Fields]) OR "cardiopulmonary resuscitation"[All Fields] OR "cpr"[All Fields]))) AND ((("mechanical"[All Fields] OR "mechanically"[All Fields] OR "mechanicals"[All Fields] OR "mechanics"[MeSH Terms] OR "mechanics"[All Fields] OR "mechanic"[All Fields]) AND ("chested"[All Fields] OR "thorax"[MeSH Terms] OR "thorax"[All Fields] OR "chest"[All Fields] OR "chests"[All Fields]) AND ("compress"[All Fields] OR "compressed"[All Fields] OR "compresses"[All Fields] OR "compressibilities"[All Fields] OR "compressibility"[All Fields] OR "compressible"[All Fields] OR "compressing"[All Fields] OR "compression"[All Fields] OR "compression s"[All Fields] OR "compressions"[All Fields] OR "compressive"[All Fields] OR "compressively"[All Fields])) OR (("mechanical"[All Fields] OR "mechanically"[All Fields] OR "mechanicals"[All Fields] OR "mechanics"[MeSH Terms] OR "mechanics"[All Fields] OR "mechanic"[All Fields]) AND ("cardiopulmonary resuscitation"[MeSH Terms] OR ("cardiopulmonary"[All Fields] AND "resuscitation"[All Fields]) OR "cardiopulmonary resuscitation"[All Fields] OR "cpr"[All Fields])) OR (("mechanical"[All Fields] OR "mechanically"[All Fields] OR "mechanicals"[All Fields] OR "mechanics"[MeSH Terms] OR "mechanics"[All Fields] OR "mechanic"[All Fields]) AND ("cardiopulmonary resuscitation"[MeSH Terms] OR ("cardiopulmonary"[All Fields] AND "resuscitation"[All Fields]) OR "cardiopulmonary resuscitation"[All Fields])) OR "LUCAS"[All Fields] OR "LUCAS-1"[All Fields] OR "LUCAS-2"[All Fields] OR "LUCAS-3"[All Fields] OR "AutoPulse"[All Fields] OR "CardioPump"[All Fields]) AND ("out of hospital cardiac arrest"[MeSH Terms] OR ("out of hospital"[All Fields] AND "cardiac"[All Fields] AND "arrest"[All Fields]) OR "out of hospital cardiac arrest"[All Fields] OR ("out"[All Fields] AND "hospital"[All Fields] AND "cardiac"[All Fields] AND "arrest"[All Fields]) OR "out of hospital cardiac arrest"[All Fields] OR "OHCA"[All Fields] OR ("heart arrest"[MeSH Terms] OR ("heart"[All Fields] AND "arrest"[All Fields]) OR "heart arrest"[All Fields] OR ("cardiac"[All Fields] AND "arrest"[All Fields]) OR "cardiac arrest"[All Fields])) AND ("outcome"[All Fields] OR "outcomes"[All Fields] OR "prognos"[All Fields] OR ("return of spontaneous circulation"[MeSH Terms] OR ("return"[All Fields] AND "spontaneous"[All Fields] AND "circulation"[All Fields]) OR "return of spontaneous circulation"[All Fields]) OR "rosc"[All Fields] OR (("mortality"[MeSH Subheading] OR "mortality"[All Fields] OR "survival"[All Fields] OR "survival"[MeSH Terms] OR "survivability"[All Fields] OR "survivable"[All Fields] OR "survivals"[All Fields] OR "survive"[All Fields] OR "survived"[All Fields] OR "survives"[All Fields] OR "surviving"[All Fields]) AND ("discharges"[All Fields] OR "discharging"[All Fields] OR "patient discharge"[MeSH Terms] OR ("patient"[All Fields] AND "discharge"[All Fields]) OR "patient discharge"[All Fields] OR "discharge"[All Fields] OR "discharged"[All Fields])) OR (("mortality"[MeSH Subheading] OR "mortality"[All Fields] OR "survival"[All Fields] OR "survival"[MeSH Terms] OR "survivability"[All Fields] OR "survivable"[All Fields] OR "survivals"[All Fields] OR "survive"[All Fields] OR "survived"[All Fields] OR "survives"[All Fields] OR "surviving"[All Fields]) AND ("hospital s"[All Fields] OR "hospitalisation"[All Fields] OR "hospitalization"[MeSH Terms] OR "hospitalization"[All Fields] OR "hospitalised"[All Fields] OR "hospitalising"[All Fields] OR "hospitality"[All Fields] OR "hospitalisations"[All Fields] OR "hospitalizations"[All Fields] OR "hospitalize"[All Fields] OR "hospitalized"[All Fields] OR "hospitalizing"[All Fields] OR "hospitals"[MeSH Terms] OR "hospitals"[All Fields] OR "hospital"[All Fields]) AND ("discharges"[All Fields] OR "discharging"[All Fields] OR "patient discharge"[MeSH Terms] OR ("patient"[All Fields] AND "discharge"[All Fields]) OR "patient discharge"[All Fields] OR "discharge"[All Fields] OR "discharged"[All Fields])) OR ("mortality"[MeSH Subheading] OR "mortality"[All Fields] OR "survival"[All Fields] OR "survival"[MeSH Terms] OR "survivability"[All Fields] OR "survivable"[All Fields] OR "survivals"[All Fields] OR "survive"[All Fields] OR "survived"[All Fields] OR "survives"[All Fields] OR "surviving"[All Fields]) OR ("mortality"[MeSH Terms] OR "mortality"[All Fields] OR "mortalities"[All Fields] OR "mortality"[MeSH Subheading])) | PUBMED | 274 |
| (manual chest compression OR manual CPR OR manual cardiopulmonary resuscitation OR conventional CPR) AND (mechanical chest compression OR mechanical cpr OR mechanical cardiopulmonary resuscitation OR LUCAS OR LUCAS-1 OR LUCAS-2 OR LUCAS-3 OR AutoPulse OR CardioPump) AND (out-of-hospital cardiac arrest OR OHCA OR cardiac arrest) AND (outcome OR prognos OR return of spontaneous circulation OR rosc OR survival to discharge OR survival to hospital discharge OR survival OR mortality) | COCHRANE LIBRARY | 75 |
| ((“manual chest compression” OR “manual CPR” OR “manual cardiopulmonary resuscitation” OR “conventional CPR”) AND (“mechanical chest compression” OR “mechanical cpr” OR “mechanical cardiopulmonary resuscitation” OR “LUCAS” OR “LUCAS-1” OR “LUCAS-2” OR “LUCAS-3” OR “AutoPulse” OR “CardioPump”) AND (“out-of-hospital cardiac arrest” OR “OHCA” OR “cardiac arrest”) AND (“outcome” OR “prognos” OR “return of spontaneous circulation” OR “ROSC” OR “survival to discharge” OR “survival to hospital discharge” OR “survival” OR “mortality”)) | SCOPUS | 251 |
