# Supplementary material for: Comparison of manual chest compression versus mechanical chest compression for out-of-hospital cardiac arrest: A systematic review and meta-analysis
Source: Medicine (Baltimore). 2024 Feb 23;103(8):e37294. doi: 10.1097/MD.0000000000037294 (PMC10883626; doi:10.1097/MD.0000000000037294)
Supplement: Supplementary file 2 [file medi-103-e37294-s002.docx]

| Type of RCT | Study | D1 | D1b | D2 | D3 | D4 | D5 | Overall bias |
| --- | --- | --- | --- | --- | --- | --- | --- | --- |
| Cluster RCT | Anantharaman 2017 | Some concerns | Low | Some concerns | Low | Low | Low | High |
| Cluster RCT | Perkin 2014 | Low | Low | Some concerns | Low | Low | Low | Some Concerns |
| Cluster RCT | Hallstrom 2006 | Some concerns | Low | Low | Low | Low | Low | Some concerns |
| Cluster RCT | Goa 2016 | High | Some Concerns | Low | Low | Low | Low | High |
| Individual RCT | Rubertsson 2015 | Low | _ | Low | Low | Low | Low | Low |
| Individual RCT | Wik 2014 | Low | _ | Low | Low | Low | Low | Low |
| Individual RCT | Smekal (2011) | Low | _ | Some Concerns | Low | Low | Low | Some Concerns |
| Individual RCT | Günaydın (2015) | Some concerns | _ | Low | Low | Low | Low | Some Concerns |

*D1: Risk of bias arising from the randomization process.*

*D1b: Risk of bias arising from the timing of identification or recruitment of participants in a cluster-randomized trial.*

*D2: Risk of bias due to deviations from the intended interventions.*

*D3: Risk of bias due to missing outcome data.*

*D4: Risk of bias in the measurement of the outcome*

*D5: Risk of bias in the selection of the reported result*
