# Supplementary material for: Comparison of manual chest compression versus mechanical chest compression for out-of-hospital cardiac arrest: A systematic review and meta-analysis
Source: Medicine (Baltimore). 2024 Feb 23;103(8):e37294. doi: 10.1097/MD.0000000000037294 (PMC10883626; doi:10.1097/MD.0000000000037294)
Supplement: Supplementary file 3 [file medi-103-e37294-s003.docx]

| Cohort Studies | Representativeness of intervention cohort | Selection of non-intervention cohort | Ascertainment of outcome | Demonstration of outcome | Comparability of cohorts on the basis of the design or analysis | Assessment of outcome | Was follow-up long enough for outcomes to occur | Adequacy of follow-up of cohorts | Final Score |
| --- | --- | --- | --- | --- | --- | --- | --- | --- | --- |
| Newberry (2018) | 1 | 1 | 1 | 1 | 1 | 1 | 1 | 1 | 8/9 |
| Ong (2006) | 1 | 1 | 1 | 1 | 1 | 1 | 1 | 1 | 8/9 |
| Zeiner (2015) | 0 | 1 | 1 | 1 | 1 | 1 | 1 | 1 | 7/9 |
| Lin (2014) | 0 | 1 | 1 | 1 | 2 | 1 | 1 | 1 | 8/9 |
| Chen (2021) | 0 | 1 | 1 | 1 | 1 | 1 | 1 | 1 | 7/9 |
| Gonzales (2018) | 1 | 1 | 1 | 1 | 1 | 1 | 1 | 1 | 8/9 |
| Savastano (2019) | 1 | 1 | 1 | 1 | 2 | 1 | 1 | 1 | 9/9 |
| Mistraletti (2022) | 1 | 1 | 1 | 1 | 1 | 1 | 1 | 1 | 8/9 |
| Tantarattanapong (2022) | 0 | 1 | 1 | 1 | 1 | 1 | 1 | 1 | 7/9 |
| Mastenbrook (2020) | 1 | 1 | 1 | 1 | 1 | 1 | 1 | 1 | 8/9 |
| Hayashida (2017) | 1 | 1 | 1 | 1 | 1 | 1 | 1 | 1 | 8/9 |
| Satterlee (2013) | 0 | 1 | 1 | 1 | 0 | 1 | 1 | 1 | 6/9 |
| Buckler (2016) | 1 | 1 | 1 | 1 | 1 | 1 | 1 | 1 | 8/9 |
| Case-Control Studies | **Adequate case definition** | **Representativeness of cases** | **Selection of control** | **Definition of control** | **Comparability of cases and controls on the basis of the design or analysis** | **Ascertainment of exposure** | **Same method of ascertainment for cases and controls** | **Non-Response rate** | **Final Score** |
| Jennings (2012) | 1 | 1 | 1 | 1 | 1 | 1 | 1 | 1 | 8/9 |
| Casner (2005) | 1 | 1 | 1 | 1 | 1 | 1 | 1 | 1 | 8/9 |
