# Supplementary material for: Comparison of manual chest compression versus mechanical chest compression for out-of-hospital cardiac arrest: A systematic review and meta-analysis
Source: Medicine (Baltimore). 2024 Feb 23;103(8):e37294. doi: 10.1097/MD.0000000000037294 (PMC10883626; doi:10.1097/MD.0000000000037294)
Supplement: Supplementary file 4 [file medi-103-e37294-s004.docx]

| Study | D1 | D2 | D3 | D4 | D5 | D6 | D7 | Overall |
| --- | --- | --- | --- | --- | --- | --- | --- | --- |
| Axelsson 2006 | Moderate | Low | Low | Low | Low | Low | Low | Moderate |

*D1: Bias due to confounding*

*D2: Bias in the selection of participants in the study*

*D3: Bias in the classification of interventions*

*D4: Bias due to deviations from intended interventions*

*D5: Bias due to missing data*

*D6: Bias in the measurement of outcomes*

*D7: Bias in the selection of the reported result*
